# Examining the Relationships Between Indoor Environmental Quality Parameters Pertaining to Light, Noise, Temperature, and Humidity and the Behavioral and Psychological Symptoms of People Living With Dementia: Scoping Review

**DOI:** 10.2196/56452

**Published:** 2024-08-09

**Authors:** Wan-Tai M Au-Yeung, Lyndsey Miller, Chao-Yi Wu, Zachary Beattie, Michael Nunnerley, Remonda Hanna, Sarah Gothard, Katherine Wild, Jeffrey Kaye

**Affiliations:** 1 Department of Neurology Oregon Health & Science University Portland, OR United States; 2 Department of Neurology Massachusetts General Hospital Harvard Medical School Boston, MA United States; 3 Department of Psychology Portland State University Portland, OR United States; 4 Fariborz Maseeh Department of Mathematics and Statistics Portland State University Portland, OR United States

**Keywords:** dementia, behavioral and psychological symptoms of dementia, neuropsychiatric symptoms, physical environment, light, noise, temperature, humidity

## Abstract

**Background:**

A common challenge for individuals caring for people with Alzheimer disease and related dementias is managing the behavioral and psychological symptoms of dementia (BPSD). Effective management of BPSD will increase the quality of life of people living with dementia, lessen caregivers’ burden, and lower health care cost.

**Objective:**

In this review, we seek to (1) examine how indoor environmental quality parameters pertaining to light, noise, temperature, and humidity are associated with BPSD and how controlling these parameters can help manage these symptoms and (2) identify the current state of knowledge in this area, current gaps in the research, and potential future directions.

**Methods:**

Searches were conducted in the CINAHL, Embase, MEDLINE, and PsycINFO databases for papers published from January 2007 to February 2024. We searched for studies examining the relationship between indoor environmental quality parameters pertaining to light, noise, temperature, and humidity and BPSD.

**Results:**

A total of 3123 papers were identified in the original search in October 2020. After an additional 2 searches and screening, 38 (0.69%) of the 5476 papers were included. Among the included papers, light was the most studied environmental factor (34/38, 89%), while there were fewer studies (from 5/38, 13% to 11/38, 29%) examining the relationships between other environmental factors and BPSD. Of the 38 studies, 8 (21%) examined multiple indoor environmental quality parameters. Subjective data were the only source of environmental assessments in 6 (16%) of the 38 studies. The findings regarding the relationship between agitation and light therapy are conflicted, while the studies that examined the relationship between BPSD and temperature or humidity are all observational. The results suggest that when the environmental factors are deemed overstimulating or understimulating for an individual with dementia, the behavioral symptoms tend to be exacerbated.

**Conclusions:**

The findings of this scoping review may inform the design of long-term care units and older adult housing to support aging in place. More research is still needed to better understand the relationship between indoor environmental quality parameters and BPSD, and there is a need for more objective measurements of both the indoor environmental quality parameters and behavioral symptoms. One future direction is to incorporate objective sensing and advanced computational methods in real-time assessments to initiate just-in-time environmental interventions. Better management of BPSD will benefit patients, caregivers, and the health care system.

## Introduction

### Background

Alzheimer disease and related dementias (ADRD) are a major public health challenge. The number of Americans aged ≥65 years with Alzheimer disease was estimated to be 6.7 million in 2023 [1]. In 2023, total payments for health care, long-term care, and hospice services for people aged ≥65 years with dementia were estimated to be US $345 billion in the United States. The number of people with Alzheimer disease in the United States is projected to grow to 13.8 million by 2060 [1].

A major component of the high psychological and financial costs of ADRD is related to addressing the needs of people living with dementia who have behavioral and psychological symptoms of dementia (BPSD), also called neuropsychiatric symptoms. BPSD encompass the challenging behaviors exhibited by people living with dementia that comprise a variety of symptoms that commonly include, but are not limited to, apathy, anxiety, depression, agitation, delusions, hallucinations, motor disturbances, and sleep changes. These symptoms are associated with faster disease progression [2], increased caregiving burden [3], and earlier placement into long-term care settings [4]. Pharmacological intervention is often not effective and is associated with undesirable side effects [5], with nonpharmacological therapy being a first-line approach to management [6].

The causes of BPSD are multiple, ranging from intrinsic neuropathologic factors to human factors such as caregiver interactions and the social-environmental milieu [7]. There is considerable evidence suggesting that environmental factors can affect the progression of different diseases as well as human behaviors [8,9]. The environment as a risk factor for ADRD has been reviewed, suggesting links between increased risks of ADRD and factors such as poor air quality, environmental toxins, and occupation-related exposures [10,11]. However, what has been less clearly examined is the potential direct relationship between person- or residence-level experienced environmental factors and contemporaneous behavioral changes associated with ADRD. This is important because most older people and people living with dementia spend the majority of their time indoors [12].

There has long been debate regarding the definition of the environment [13]. For the purpose of this review, we are focusing on the ambient environment as defined by Harris et al [14]. Factors pertaining to the ambient environment, such as light, noise, temperature, and humidity, contribute to the visual, acoustic, and thermal comforts of the occupants and may be associated with their BPSD [15]; for example, inadequate light has been suggested to be a risk factor for sundowning syndrome (people with late-stage dementia exhibiting more agitated behaviors in the late afternoon and evening) because of the role of light in vision and its role in circadian rhythm modulation [16,17]. Therefore, designing an indoor environment that would address the needs of people living with dementia is paramount and may reduce their BPSD. A first step to creating an optimal indoor environment for people living with dementia with BPSD is to understand which environmental factors are related to BPSD and how they impact the symptoms.

Previous papers have reviewed the effect of the long-term care environment on physical activity levels [18], the achievement of therapeutic goals such as safety and socialization [19], and neuropsychiatric symptoms [20,21]. However, these reviews have primarily focused on the parameters of the built environment (eg, size and layout of spaces), interior design, and occupancy and staffing ratios, rather than aspects of the environmental quality that impact comfort, such as temperature and humidity, noise levels, and lighting. Comfort is especially important to consider for people living with dementia because BPSD represent an expression of discomfort, stress, and unmet needs when insight and communication become more difficult [22]. Nevertheless, anosognosia and aphasia also prevent the subjective measurement of comfort. Thus, it is necessary to understand the impact of various objective aspects of environmental quality that may improve comfort and, in turn, BPSD.

### Objectives

Accordingly, we conducted this scoping review to (1) examine how indoor environmental quality parameters pertaining to light, noise, temperature, and humidity are associated with BPSD and how controlling these parameters can help manage these symptoms and (2) identify the current state of knowledge on the relationship between indoor environmental quality parameters and BPSD, current gaps in the research, and potential future directions.

## Methods

### Overview

We followed the PRISMA-ScR (Preferred Reporting Items for Systematic Reviews and Meta-Analyses extension for Scoping Reviews) reporting guidelines for this scoping review. We conducted a scoping review because we aimed to (1) provide an overview of the broad literature examining the relationship between indoor environmental quality parameters and BPSD and (2) identify gaps and future directions.

The databases used were CINAHL, Embase, MEDLINE, and PsycINFO. A literature search strategy was crafted for each database that would retrieve records containing a combination of appropriate index terms and text words pertaining to the following concepts: dementia (dementia, Alzheimer disease, etc), environmental conditions (heat, temperature, light, humidity, etc), and BPSD (anxiety, depression, apathy, agitation, pacing, etc). The complete search strategies can be found in Multimedia Appendices 1-3. The original search was performed on October 7, 2020, and updated on April 21, 2022, and February 22, 2024. The inclusion and exclusion criteria are presented in Textbox 1.

Six reviewers (WMA-Y, CW, MN, LM, RH, and KW) completed the screening process, which consisted of two stages: (1) title and abstract screening and (2) full-text screening. First, the title and abstract of each paper were reviewed by 2 reviewers independently to examine whether the studies met the inclusion criteria. The reviewers then met iteratively to reconcile any differences in their decisions regarding whether a particular paper should be included. If the 2 reviewers could not come to an agreement, a third reviewer would help reach a decision. After title and abstract screening was completed, the included papers at this stage were retrieved for full-text screening, and the process was repeated after reading the papers in their entirety.

**Inclusion and exclusion criteria.**

**Inclusion criteria**

- Written in English
- Peer reviewed
- Studies of older adults with Alzheimer disease and related dementias (ADRD), not including mild cognitive impairment
- Relationships between indoor environmental quality parameters, including light, noise, temperature, and humidity and the behaviors of people living with dementia
- Exposure to environmental conditions leading to behavioral change
- Observational or controlled
- All residential settings
- Studies using measurements of people’s behaviors and psychological symptoms as 1 of the outcomes
- Studies that include measurements or descriptions of the indoor environmental quality parameters (light, noise, temperature, and humidity)

**Exclusion criteria**

- Review articles, commentaries, opinions, theses, and letters to editor
- Studies on genetics as risk factors for ADRD
- Studies that examine the effect of decoration, furniture arrangement, and home-like feeling on participants’ behaviors
- Studies on the environment as a risk factor for developing ADRD
- Studies on horticultural therapy, music therapy, multisensory stimulation therapy, virtual or augmented reality, or outdoor activities
- Studies published before 2006 (the 15-year cutoff was chosen to include articles that best represent the current state of knowledge)

Six reviewers (WMA-Y, MN, ZB, LM, RH, and SG) extracted data from the included papers after the screening process was completed. A survey built in Qualtrics (Qualtrics International Inc) was created to assist this process. The survey listed questions covering study design, year of publication, the region and residential settings in which the research was conducted, sample size, demographics of the sample, which environmental conditions were assessed and how they were examined, and which BPSD were evaluated and how they were measured. Next, the papers were categorized by the examined environmental factor or factors: (1) light level, (2) noise level, (3) temperature or humidity, and (4) multiple indoor environmental quality parameters. If there were unified themes of BPSD examined in the articles (eg, agitation and mood-related symptoms), the study results of each theme would be synthesized by summarizing the counts of different study designs and providing a narrative summary [23]. All study data were also summarized into tables. In doing so, the current state of knowledge, gaps in research, and future directions were identified.

### Ethical Considerations

Institutional review board approval was not required for this scoping review because the research did not involve human participants.

## Results

### Overview

**Figure 1:**
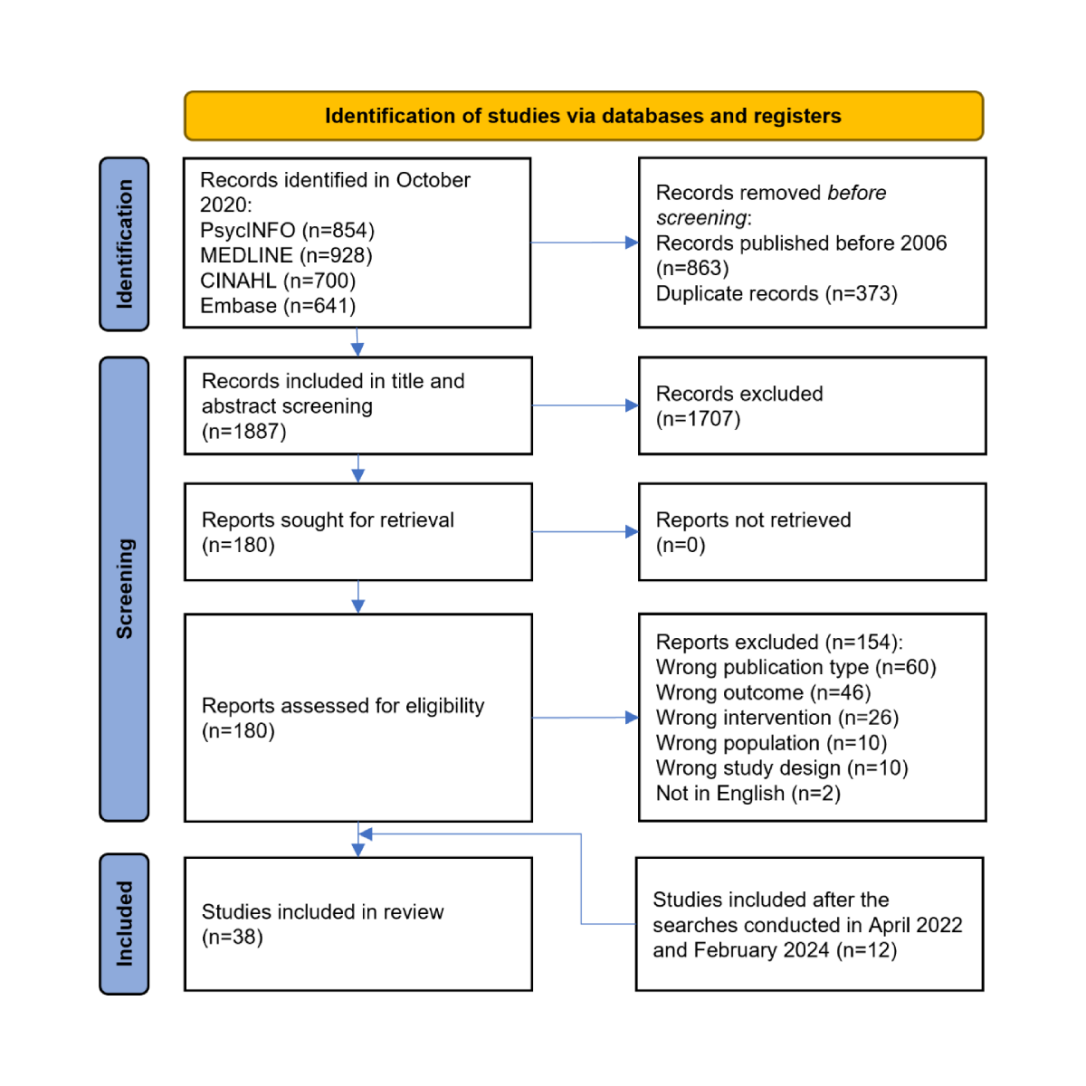
PRISMA (Preferred Reporting Items for Systematic Reviews and Meta-Analyses) flow diagram of the search and screening results.

A total of 3123 articles were identified in the search initiated on October 7, 2020. After excluding papers published before 2006 as well as duplicates, the titles and abstracts of 1887 articles were screened, after which 1707 (90.46%) articles were excluded, and 180 (9.54%) were retrieved for full-text screening. Ultimately, of these 180 articles, 26 (14.4%) were included. The search was updated on April 21, 2022, and on February 22, 2024, and 12 articles were added to the final pool; hence, 38 papers were included in this scoping review (Figure 1). Of the 38 papers, 8 (21%) were randomized controlled trials (RCTs), 14 (37%) were quasi-experiments, 5 (13%) were pretest-posttest studies, 6 (16%) were cross-sectional studies, 4 (11%) were case series, and 1 (3%) was a cohort study (refer to Table 1 for more information).

**Table 1:** Summary of residential settings, region, study design, intervention, sample size, participant cognitive status, and participant age for all included studies.

Study, year	Residential settings	Region	Study design	Intervention (if any)	Sample size	Participant cognitive status	Participant age (y)
Algase et al [24], 2010	Assisted living units, nursing homes	North America	Cross-sectional study	—^a^	M^b^: 28, F^c^: 94	MMSE^d^ score: mean 7.4 (SD 7.2)	Mean 83.7 (SD 6.48; range 68-102)
Bankole et al [25], 2020	Personal homes	North America	Case report or series	—	M: 6, F: 6 (the 12 participants lived with primary caregivers)	All participants had dementia	Mean 79.67 (SD 7.5; range 68-92)
Barrick et al [26], 2010	Memory care units, psychiatric hospital	North America	Quasi-experiment	Four lighting conditions were presented during multiple 3-week intervention periods: bright light in the morning (7-11 Am), bright light in the evening (4-8 PM), bright light all day (7 AM-8 PM), and standard lighting (ie, the baseline condition); bright light was administered at 2000 to 3000 lux, while standard lighting was administered at 500 to 600 lux	M: 35, F: 31	Cognitive status was measured using the MDS-COGS^e^ and MMSE; the distribution of cognitive impairment severity among participants was as follows: mild=3, moderate=18, severe=31, and very severe=14	North Carolina, United States: <65=6 (13%), 65-79=24 (52%), and ≥80=16 (35%); Oregon, United States: <65=0 (0%), 65-79=4 (20%), and ≥80=16 (80%)
Bautrant et al [27], 2019	Nursing homes	Europe	Pretest-posttest study	Skylike ceiling tiles in part of the shared premises, gradual decrease of the illuminance at night, soothing streaming music, reinforcement of the illuminance during the day, walls painted in light beige, oversized clocks in corridors, and night team clothes color (dark blue) different from that of the day team (sky blue)	M: 2, F: 17	Severe dementia; MMSE scores were <15 for all patients	≥65; mean 86.3 (SD 5.4)
Bicket et al [28], 2010	Assisted living units	North America	Cross-sectional study	—	M: 80, F: 246	Demented: 194, nondemented: 131	Demented: mean 86.1 (SD 8.1), nondemented: mean 84.3 (SD 9.6)
Bromundt et al [29], 2019	Nursing homes	Europe	Quasi-experiment (balanced crossover, within-participant study)	This was a 17-week trial with a balanced crossover, within- participant study design; the intervention involved exposure to individually timed dawn-dusk simulator over participants’ beds for 7-8 weeks	M: 3, F: 17	S-MMSE^f^ score: mean 13.15 (SD 10.30); S-MMSE score at baseline: mean 14.35 (SD 10.51), S-MMSE score at the end of the study: mean 11.95 (SD 11.56)	Mean 85.6 (SD 5.8)
Burns et al [30], 2009	Nursing homes	Europe	Randomized controlled trial	Full-spectrum bright light therapy (10,000 lux) for 2 hours from 10 AM to noon for 2 weeks; the control group was administered standard fluorescent tube light (100 lux) for 2 hours from 10 AM to noon for 2 weeks	M: 16, F: 32	MMSE score at baseline: 5.1 for the standard lighting therapy group and 6.9 for the bright light therapy group	Standard light therapy group: mean 82.5 (SD 1.5), bright light therapy group: mean 84.5 (SD 1.7)
Cohen-Mansfield et al [31], 2010	Nursing homes	North America	Quasi-experiment (indoor environmental quality parameters were not manipulated)	Presentation of 9 categories of stimuli: live human social, real pet, simulated social, self-identity, inanimate social, music, manipulative, reading, and task- or work-related	M: 42, F: 151	MMSE score: mean 7.2 (SD 6.3; range 0-23)	Mean 86 (range 60-101)
Cohen-Mansfield et al [32], 2011	Nursing homes	North America	Quasi-experiment (indoor environmental quality parameters were not manipulated)	Presentation of 9 categories of stimuli: live human social, real pet, simulated social, self-identity, inanimate social, music, manipulative, reading and task-or work-related	M: 42, F: 151	MMSE score: mean 7.2 (SD 6.3; range 0-23)	Mean 86 (range 60-101)
Cohen-Mansfield et al [33], 2012	Nursing homes	North America	Quasi-experiment (indoor environmental quality parameters were not manipulated)	Presentation of 9 categories of stimuli: live human social, real pet, simulated social, self-identity, inanimate social, music, manipulative, reading and task-or work-related	M: 42, F: 151	MMSE score: mean 7.2 (SD 6.3; range 0-23)	Mean 86 (range 60-101)
Cohen-Mansfield [34], 2020	Nursing homes	North America	Cross-sectional study	—	M: 26, F: 43	CPS^g^ score: mean 4.19	Mean 86.58 (SD 7.93)
Dowling et al [35], 2007	Nursing homes	North America	Randomized controlled trial	During either morning (9:30-10:30 AM) or afternoon (3:30-4:30 PM), bright light (>2500 lux in gaze direction) was administered Monday through Friday for 10 weeks; Brite Lite IV (Apollo Light Systems, Inc) light boxes were used when necessary to supplement the ambient light	M: 13, F: 57	MMSE score: mean 7 (SD 7; range 0-23)	Mean 84 (SD 10; range 58-98)
Figueiro et al [36], 2014	Nursing homes	North America	Pretest-posttest study	Low-level *bluish-white* lighting designed to deliver high circadian stimulation during the daytime was installed in 14 nursing home resident rooms for 4 weeks	M: 5, F: 9	BIMS^h^ score: mean 7.7 (SD 2.3)	Mean 86.9 (SD 4.4)
Figueiro et al [37], 2019	Assisted living units, long-term care units	North America	Randomized controlled trial	Crossover design clinical trial administered an all-day active or control lighting intervention for two 4-week periods (separated by a 4-week washout); the active lighting intervention provided high circadian stimulus, and the control intervention provided low circadian stimulus that was below the threshold for activation of the circadian system	M: 16, F: 30	MMSE score between 4 and 24 or BIMS score between 3 and 12	Mean 85.1 (SD 7.1)
Figueiro et al [38], 2020	Assisted living units, long-term care units	North America	Pretest-posttest study	Participants were exposed to a single daytime (from approximately 6 AM-8 AM to 6 PM) tailored lighting intervention that provided high levels of circadian-effective light; the light-delivery method (ie, custom-built floor lamps, light boxes, and light tables) was dependent on where individual participants spent most of their day	M: 20, F: 27	MMSE scores: F=mean 15.2 (SD 3.4) and M=mean 13.6 (SD 2.9); BIMS scores: F=mean 4.0 (SD 1.7) and M=mean 5.6 (SD 2.9)	Mean 85.3 (SD 7.1)
Figueiro et al [39], 2023	Assisted living units, memory care units	North America	Quasi-experiment	With a crossover, placebo-controlled design, 3 different lighting modes (light tables, light trays, and ambient room lighting) were used to deliver high levels of circadian stimulus to the participants’ eyes for two 8-week intervention periods in a counter balanced order with a 4-week washout between the study’s 2 conditions (dim light control vs active intervention)	M: 3, F: 11	Moderate to severe ADRD^i^ indicated by a GDS^j^ score of 4.0 to 6.0; the mean BCRS^k^ score was 4.83 (SD 1.01), and the mean GDS score was 4.89 (SD 1.1)	Mean 84.1 (SD 8.9)
Garre-Olmo et al [40], 2012	Nursing homes	Europe	Cross-sectional study	—	M: 37, F 123	MMSE score: mean 4.1 (SD 6.3); Barthel Index score: mean 10.5 (SD 19.3)	Mean 82.6 (SD 11.6)
Hickman et al [41], 2007	Memory care units, specialized older adult center	North America	Quasi-experiment	Ambient bright light therapy was delivered through a high-intensity, low-glare lighting system installed in the public areas that involved 4 lighting conditions: morning bright light, evening bright light, all-day bright light, and standard lighting. The bright light conditions were delivered at 2000 to 2500 lux, while the standard lighting condition was delivered at 500 to 600 lux	M: 35, F: 31	Mild to moderate dementia: M=12 (34.3%), F=9 (29%); severe dementia: M=15 (42.9%), F=16 (51.6%); very severe dementia: M=8 (22.9%), F=6 (19.4%)	<65: M=5 (14.3%), F=1 (3.2%); 65-79: M=18 (51.4%), F=10 (32.3%); ≥80: M=12 (34.3%), F=20 (64.5%)
Hjetland et al [42], 2021	Nursing homes	Europe	Randomized controlled trial	A 24-week cluster-randomized, placebo-controlled trial with an intervention involving ambient light administered at 1000 vertical lux and 6000 K from 10 AM to 3 PM, with gradually increasing and decreasing light levels before and after this interval, delivered by Glamox AS	M: 22, F: 47	MMSE score: mean 6.4 (SD 6.7)	Mean 83.5 (SD 7.1)
Jao et al [43], 2015	Assisted living units, nursing homes	North America	Case report or series	—	M; 9, F: 29	MMSE score: mean 12.9 (SD 6.5; range 2-23)	Mean 82.7 (SD 6.3; range 68-94)
Joosse [44], 2011	Nursing homes	North America	Cross-sectional study	—	M: 11, F: 42	MMSE score: mean 6.83 (SD 6.67; range 0 to 19); specifically, 37 (70%) scored <13 (severe dementia), and 16 (30%) scored 13 to 20 (moderate dementia)	Mean 86.53 (SD 9.32; range 61 to 103)
Kim et al [45], 2021	Dementia clinic	Asia	Quasi-experiment	The treatment group (14 participants) sat approximately 60 cm away from a small (136×73×16 mm) light-emitting diode light box for 1 hour each in the morning for 2 weeks, and the control group (11 participants) wore dark, blue-attenuating sunglasses during the 1-hour exposures; the morning light exposure started 9 to 10 hours after each individual’s dim light melatonin onset	M: 7, F: 18	MMSE-KC^l^: treatment group (M: 2, F: 12)=mean 16.36 (SD 5.09), control group (M: 2, F: 12)=mean 16.90 (SD 4.91)	Treatment group: mean 77.36 (SD 5.79), control group: mean 78.55 (SD 7.71)
Kolberg et al [46], 2021	Nursing home dementia units	Europe	Randomized controlled trial	A 24-week cluster-randomized, placebo-controlled trial with an intervention involving ambient light administered at 1000 lux and 6000 K from 10 AM to 3 PM, with gradually increasing and decreasing light levels before and after this interval	M: 22, F: 47	MMSE score: median 4.0 (IQR 1.0-9.2)	Median 85.0 (IQR 79.0-88.0)
Konis et al [47], 2018	Dementia care communities	North America	Quasi-experiment	The daylight exposure of participants was increased by taking them to the perimeter zone of a daylit room from 8 AM to 10 AM for socialization over a period of 12 weeks; the perimeter zone was the region of the room within 3 m from the windows; it was administered every day throughout the study	M: 21, F: 56	Alzheimer disease: daylight group=17 (37%) patients, control group=8 (25.8%) patients; frontotemporal dementia: daylight group=1 (2.2%) patient, control group=1 (3.2%) patient; Lewy body dementia: daylight group=0 (0%) patients, control group=1 (3.2%) patient; vascular dementia: daylight group=2 (4.3%) patients, control group=1 (3.2%) patient; dementia not otherwise specified: daylight group=19 (41.3%) patients, control group=17 (54.8%) patients; mild cognitive impairment: daylight group: 6 (13%) patients, control group=2 (6.5%) patients; not specified: daylight group=1 (2.2%) patient, control group=1 (3.2%) patient	Mean 85.3 (SD 7.0)
Lee et al [48], 2016	Long-term care units (Richmond Manor and Maple Manor)	North America	Cohort study	—	M: 6, F: 6	Early or middle stage of ADRD	Richmond Manor: mean 82.9 (SD 8.9), Maple Manor: mean 77.6 (SD 9.8)
Lin et al [49], 2018	Dementia care centers	Asia	Quasi-experiment	Experimental group participants received 20 minutes of white noise consisting of ocean, rain, wind, and running water sounds between 4 PM and 5 PM daily over a period of 4 weeks, with volume maintained at 55-70 dB; the comparison group received routine care	M: 23, F: 40	NR^m^	Experimental group: mean 81.57 (SD 10.52), comparison group: mean 79.29 (SD 8.41)
Liu et al [50], 2021	Nursing homes, communities	Asia	Quasi-experiment	Participants in the experimental group were exposed to ambient light at 2500 lux for at least 60 minutes per day from 9 AM to 10 AM, Monday through Friday, over 8 weeks	M: 7, F: 28	Mild dementia: 10, moderate dementia: 16, severe dementia: 9	60-95
Liu et al [51], 2022	Nursing homes	Asia	Quasi-experiment	Participants in the experimental group were exposed to ambient light at 2500 lux for at least 60 minutes per day from 9 AM to 10 AM, Monday through Friday, over 8 weeks	M: 7, F: 28	Mild dementia: 10, moderate dementia: 16, severe dementia: 9	60-95 experimental group: mean 83.9 (SD 7.1), comparison group: mean 80.2 (SD 7.2)
McCurry et al [52], 2011	Personal homes	North America	Randomized controlled trial	Intervention 1: participants attempted to reach the goal of walking for 30 minutes continuously per day; intervention 2: participants sat in front of a light box (SunRay; The SunBox Company) for 1 hour per day, timed to be within 2-hour window before the participants’ habitual bedtime; intervention 3: combination treatment comprising walking, light exposure, and guided sleep education (NITE-AD^n^)	M: 59, F: 73	MMSE scores: walking group=mean 19.2 (SD 7.7); light group=mean 17.9 (SD 7.0), NITE-AD group=mean 19.1 (SD 5.8), control group=mean 18.7 (SD 6.9)	Walking group: mean 82.2 (SD 8.5), light group: mean 80.6 years (SD 7.3), NITE-AD group: mean 80.0 (SD 8.2), control group: mean 81.2 (SD 8.0)
Olsen et al [53], 2016	Personal homes, nursing homes	Europe	Cross-sectional study	—	M: 67, F: 126	Nursing home residents (Clinical Dementia Rating Scale): mild dementia=9%, moderate dementia=43.6%, severe dementia=47.4%; home-dwelling people with dementia (Clinical Dementia Rating Scale): mild dementia=43.5%, moderate dementia=47%, severe dementia=5.2%	Nursing home residents: mean 84.6 years (SD 6.50), home-dwelling people with dementia: mean 82.6 (SD 6.84)
Onega et al [54], 2016	Long-term care units	North America	Randomized controlled trial	Individuals were seated approximately 27 inches away from a bright light that delivered 10,000 lux of light (treatment arm) or a low-level light that delivered 250 lux of light (control) for 30 minutes twice a day 5 times a week for 8 weeks	M: 17, F: 43	MMSE scores ranged from 0 to 22; participants were classified into 3 categories based on the MMSE scores: 7 (11.7%) had mild dementia, 11 (18.3%) had moderate dementia, 42 (70%) had severe dementia	Mean 82.6 (SD 9.6)
Riemersma-van der Lek et al [55], 2008	Group care facilities	Europe	Randomized controlled trial	Random assignment by facility to long-term daily treatment with whole-day bright (approximately 1000 lux) or dim (approximately 300 lux) light and by participant to evening melatonin (2.5 mg) or placebo for a mean of 15 (SD 12) months (maximum period of 3.5 years)	M: 19, F: 170	Treatment group, placebo: MMSE score=mean 14.3 (SD 7.0); treatment group, light: MMSE score=mean 14.5 (SD 6.2); treatment group, melatonin: MMSE score=mean 15.3 (SD 5.3); treatment group, light+melatonin: MMSE score=mean 14.7 (SD 6.8)	Mean 85.8 (SD 5.5)
Saidane et al [56], 2023	Nursing homes	Europe	Pretest-posttest study	Naturalistic light systems that replicated the spectrum distribution of natural light from dusk to dawn	5	NR	NR
Sloane et al [57], 2007	Long-term care units	North America	Quasi-experiment	The study used a cluster-unit crossover experimental design to evaluate 4 ambient lighting conditions: morning (7-11)=high-intensity light, evening (4-8)=high-intensity light, all day (7 AM-8 PM)=high-intensity light, and industry minimum standard lighting	M: 35, F: 31	Mild to moderate cognitive impairment: 21 (31.8%), severe cognitive impairment: 31 (47%), very severe cognitive impairment: 14 (21.2%)	Mean 79
Son and Kwag [58], 2020	Center for dementia	Asia	Quasi-experiment	In the experimental group, a walking program with white noise was applied 3 times a week for 4 weeks; white noise was provided by a white noise generator, with volume maintained at 40 to 50 dB; in the control group, only the walking program was applied	M: 16, F: 16	NR	Range 71-80; experimental group: mean 75.06 (2.82), control group: mean 75.31 (SD 3.07)
Tartarini et al [59], 2017	Nursing homes	Oceania	Case report or series	—	M: 14, F: 7	Psychogeriatric Assessment Scale scores of ≥10	Range 61-92
Wahnschaffe et al [60], 2017	Nursing homes	Europe	Pretest-posttest study	From midwinter on, a ceiling-mounted dynamic lighting system was installed in the common room of a nursing home and programmed to produce high illuminance with higher blue light proportions during the day and lower illuminance without blue light in the evening	M: 5, F: 7	MMSE score: mean 12.1 (SD 9.2)	Mean 79.1 (SD 11)
Wahnschaffe et al [61], 2017	Nursing homes	Europe	Case report or series	—	M: 1, F: 19	*ICD-10*^o^ criteria: Alzheimer disease=9, vascular dementia=2, frontotemporal dementia=1, Korsakoff syndrome=1, and dementia subtypes (which were not further specified)=7	Mean 83.8 years (SD 8.8)

^a^Not applicable.

^b^M: male.

^c^F: female.

^d^MMSE: Mini–Mental State Examination.

^e^MDS-COGS: Minimum Data Set Cognition Scale.

^f^S-MMSE: Standardized Mini–Mental State Examination.

^g^CPS: Cognitive Performance Scale.

^h^BIMS: Brief Interview for Mental Status.

^i^ADRD: Alzheimer disease and related dementias.

^j^GDS: Global Deterioration Scale.

^k^BCRS: Brief Cognitive Rating Scale.

^l^MMSE-KC: Korean version of the MMSE developed as part of the Korean version of the Consortium to Establish a Registry for Alzheimer’s Disease assessment packet.

^m^NR: not reported.

^n^NITE-AD: Nighttime Insomnia Treatment and Education in Alzheimer’s Disease.

^o^ICD-10: International Classification of Diseases, Tenth Revision.

The following sections synthesize the findings of the included studies, categorized by the indoor environmental quality factor or factors examined (Table 2).

**Table 2:** Summary of environmental conditions and behavioral and psychological symptoms of dementia (BPSD) outcomes examined, the subjective and objective methods of assessment, and the main results for all included studies.

Study, year	Environmental condition or conditions measured or manipulated	Subjective indoor environmental quality parameter assessment (if any)	Objective indoor environmental quality parameter assessment (if any)	Behavioral or psychological outcomes	Subjective BPSD assessment (if any)	Objective BPSD assessment (if any)	Main results
Algase et al [24], 2010	Light level, noise level, temperature, humidity, environmental ambience, crowding	Ambience scale, presence or absence of people within 8 feet of the participant	Gossen Color-Pro 3F light meter (Gossen Color-Pro 3F light meter, Bogen Photo Corp), Quest Technologies Sound Level Meter Model 2400, and RadioShack Thermometer with Indoor Humidity Gauge 63-1013	Wandering	Observations from videotapes	—^a^	Brighter light, more variation in sound levels, and a higher engaging quality of the environment were associated with wandering, and a higher soothing quality of the environment was associated with periods when wandering did not occur
Bankole et al [25], 2020	Light level, noise level, temperature, humidity, atmospheric pressure	—	Off-the-shelf sensors	Agitation, depression, sleep, quality of life, overall level of BPSD	NPI-Q^b^, CMAI-C^c^, PSQI^d^	Shimmer3 sensor for people with dementia and Pebble sensor for caregivers in phase 1; Pebble for both people with dementia and caregivers in phase 2	Temperature, atmospheric pressure, and time showed strong correlations with agitation, which was self-reported by the primary caregivers
Barrick et al [26], 2010	Light level	—	—	Agitation	CMAI^e^, observational agitation using the presence or absence of 8 agitated behaviors	—	No therapeutic conditions improved agitation in comparison to standard lighting
Bautrant et al [27], 2019	Light level, skylike ceiling tiles, soothing streaming music, walls painted in light beige, oversized clocks in corridors	—	—	Agitation, wandering, screaming, episodes of BPSD	Number and duration of disruptive BPSD episodes were systematically collected	—	BPSD prevalence reduced after plain environmental rearrangements aimed at improving spatial and temporal orientation were put in place
Bicket et al [28], 2010	Light level	TESS-NH/RC^f^	—	Quality of life, overall level of BPSD, fall risk	NPI^g^	—	The Assisted Living Environmental Quality Score was positively associated with the Alzheimer’s Disease–Related Quality of Life score (*P*=.01), while it was strongly negatively associated with NPI total score (*P*<.001) and negatively correlated with fall risk (*P*=.04)
Bromundt et al [29], 2019	Light level	—	—	Agitation, mood, circadian rhythm (rest-activity pattern), activities of daily living, quality of life, alertness, verbal interaction, well-being, cheerfulness, memory, disturbing behavior	CMAI, VAS^h^	Wrist actimetry (MotionWatch 8; CamNtech)	Dawn-dusk simulator exposure led to significantly better mood in the morning hours (*P*=.002), while it did not significantly influence circadian parameters and sleep parameters (*P*>.05)
Burns et al [30], 2009	Light level	—	—	Agitation, depression, sleep	CMAI, CSDD^i^, MOUSEPAD^j^, CRBRS^k^, sleep charts	Actiwatch (supplied by CamNtech)	Results suggested that the bright light intervention had a limited effect in reducing agitation, but it improved sleep in older adults with dementia
Cohen-Mansfield et al [31] 2010	Light level, noise level, number of persons in proximity	Environment portion of ABMI^l^	—	Engagement	Observational measurement of engagement	—	Attention and engagement were the best when light level was moderate; engagement was the best when there was a moderate level of noise; attention to the engagement stimulus was significantly higher when there were 4 to 9 people in proximity in comparison to more or fewer people (*P*<.01)
Cohen-Mansfield et al [32], 2011	Light level, noise level, number of persons in proximity	Environment portion of ABMI	—	Agitation	ABMI, CMAI	—	Lighting and background noise did not affect agitation significantly, which may be due to little variation in these variables and their reliance on subjective perception scales
Cohen-Mansfield et al [33], 2012	Light level, noise level, number of persons in proximity	Environment portion of ABMI	—	Pleasure	Lawton Modified Behavior Stream	—	Pleasure was most likely to occur in environments with moderate noise levels
Cohen-Mansfield [34], 2020	Light level, noise level, temperature, location, time of day, total group size	Reported by therapeutic recreation staff	—	Mood, engagement, sleepiness	GOME^l^	—	Background noise and time of day significantly affected outcome variables after controlling for participants’ cognitive functioning and group topic; background noise was related with decreased engagement and increased sleepiness; there was little variation concerning temperature and light
Dowling et al [35], 2007	Light level	—	Cal LIGHT 400 calibrated precision light meter for monitoring light levels in gaze direction for each participant	Agitation, depression, overall level of BPSD, appetite or eating disorders, aberrant motor behavior, dysphoria	NPI-NH^n^	—	Bright light therapy was found not to clinically affect neuropsychiatric behaviors
Figueiro et al [36], 2014	Light level	—	Daysimeter worn on the wrist to estimate light exposure of each participant	Agitation, depression, circadian rhythm (rest-activity pattern), activities of daily living, sleep	CMAI, CSDD, PSQI	Daysimeter	300 to 400 lux of a bluish-white light significantly improved sleep efficiency, total sleep time, and global PSQI scores (*P*<.05) and decreased depression (CSDD) and agitation (CMAI) scores (*P*<.05)
Figueiro et al [37], 2019	Light level	—	Daysimeter as a pendant to estimate light exposure of each participant	Agitation, depression, circadian rhythm (rest-activity pattern), sleep, quality of life	CMAI, CSDD, PSQI	Actiwatch 2 (Philips Respironics)	The experimental group had significantly improved PSQI scores compared to the control group (*P*<.001); regarding secondary outcomes, the experimental group had significantly greater improvements in depression and agitation compared to the control group (*P*<.05)
Figueiro et al [38], 2020	Light level	—	Daysimeter as a pendant to estimate light exposure of each participant	Agitation, depression, mood, sleep	CMAI, CSDD, PSQI	Actiwatch 2 (Philips Respironics)	Long-term daily light exposure showed a positive correlation with reduced PSQI scores with increasing efficacy as the study went on; there were significant improvements in depression starting at around week 3; agitation also improved around week 9
Figueiro et al [39], 2023	Light level	—	—	Depression, circadian rhythm (rest-activity pattern), sleep	CSDD, PSQI, SDI^o^	Actiwatch 2 (Philips Respironics)	Under the active condition, both objective and subjective sleep improved significantly (*P*=.02)
Garre-Olmo et al [40], 2012	Light level, noise level, temperature	—	DT-8820 environment meter (Shenzhen Everbest Machinery Industry Co Ltd) in each participant’s bedroom and in the dining room and living room of each nursing home	Affect, mood, pain, quality of life	QUALID^p^, NPI-NH, PAIN-AD^q^	—	High temperature in the bedroom was associated with lower quality of life, high noise levels in the living room were associated with low social interactions, and low lighting levels in the bedroom were associated with more severe negative affective mood
Hickman et al [41], 2007	Light level	—	—	Depression	CSDD	—	The treatment had different effects on men and women; study results did not support the use of ambient bright light therapy as a treatment for depressive symptoms in persons with dementia; however, a subpopulation of persons with dementia may benefit from this intervention
Hjetland et al [42], 2021	Light level	—	—	Mood, circadian rhythm (rest-activity pattern), activities of daily living, cognition, sleep, overall level of BPSD	NPI-NH	Actiwatch 2 (Philips Respironics)	There was discrepancy in the subjective and objective sleep measures; actigraphically measured sleep outcomes showed no statistically significant differences between patients in the intervention and control groups; however, better sleep was reported using the SDI in the intervention group
Jao et al [43], 2015	Light level, noise level, environmental ambience, crowding, staff familiarity, environmental stimulation	PEAR^r^-Environment subscale	Gossen Color-Pro 3F light meter (Bogen Photo Corp) and the Quest Technologies Sound Level Meter	Apathy	PEAR-Apathy subscale	—	Among the 6 characteristics of environmental stimulation, stimulation clarity and stimulation strength were the only 2 significant factors affecting apathy scores; ambience, crowding, staff familiarity, light, and sounds did not show significant effects on apathy
Joosse [44], 2011	Noise level, number of persons in proximity, spatial environment	—	SoundPro DL 2 (Quest Technologies) sound level meter	Agitation	Wisconsin Agitation Inventory	—	After controlling for potential confounding variables of mental status, hearing impairment, and visual impairment, sound was found to contribute to agitation
Kim et al [45], 2021	Light level	—	—	Depression, sleep, overall level of BPSD	KNPI-Q^s^, CSDD-K^t^, PSQI, VAS-GV^u^, VAS-GA^v^	Actiwatch 2 (Philips Respironics)	The study findings suggest that morning blue-enriched light therapy has a benefit in improving sleep and cognitive function in patients with Alzheimer disease
Kolberg et al [46], 2021	Light level	—	—	Agitation, affect, anxiety, delusion, depression, hallucination, mood, circadian rhythm (rest-activity pattern), sleep, overall level of BPSD	CSDD, NPI-NH	—	Compared to the control group, the intervention group had a larger reduction on the composite scores of both the CSDD and the NPI-NH, as well as on the NPI-NH Affect subsyndrome and the CSDD mood-related signs subscale at follow-up after 16 weeks
Konis et al [47], 2018	Light level	—	Digital charge-coupled device spectrometer	Depression, overall level of BPSD	CSDD, NPI-NH	—	Participants in the daylight intervention experienced an average decrease over the trial in the NPI-NH and CSDD scores, while the control participants showed average but nonsignificant increases in both NPI-NH and CSDD scores; difference in outcome changes of the intervention group achieved statistical significance for the CSDD but not for the NPI-NH
Lee et al [48], 2016	Number of persons in proximity, spatial environment, social environment, crowding, light level	TESS-NH^w^	—	Mood, social engagement	MOSES^x^	—	Residents who stayed in the traditional, large-scale unit showed significantly worse decline in irritable behaviors compared to those who stayed in the small-scale, home-like unit across time; the small-scale, home-like unit had significantly better ratings in stimulation (lighting, visual and tactile stimulation, and noise) as well as personalization according to the TESS-NH
Lin et al [49], 2018	Noise level	—	TES-1350A sound level meter	Agitation	CMAI	—	The research results showed that agitated behaviors decreased significantly in the experimental group; in the comparison group, agitated behaviors decreased insignificantly
Liu et al [50], 2021	Light level	—	—	Overall level of BPSD	NPI	Accelerometer (XA-5)	The NPI scores, which were derived using generalized estimating equation with medication (benzodiazepines) as a covariate, were significantly reduced by the fifth and ninth weeks; a lower NPI score indicates less severe BPSD
Liu et al [51], 2022	Light level	—	—	Circadian rhythm (rest-activity pattern), sleep	Sleep charts	Accelerometer (XA-5)	The experimental group showed significantly improved sleep efficiency, sleep duration, and awakening time from baseline to the fifth and ninth week, which was higher than the improvement in the comparison group; the number of nighttime awakenings decreased in the experimental and comparison groups; for circadian rhythm, the experimental group showed significant improvement in sleep onset and sleep offset, which were higher than the improvements in the comparison group
McCurry et al [52], 2011	Light level	—	—	Sleep	SDI	Micro Mini Motionlogger actigraph (Ambulatory Monitoring, Inc)	This study found that exposure to light or increase in walking can have a benefit regarding nighttime sleep; this type of treatment is implementable by caregivers to increase the well-being of those in long-term care facilities; patients who adhered to treatment best saw the best results
Olsen et al [53], 2016	Light level	—	ActiSleep+ (ActiGraph)	Sleep, quality of life, social contact, activity	—	ActiSleep+ (ActiGraph)	People living with dementia in nursing homes had lower quality of life in comparison to those at home, while nursing homes were associated with lower light level; however, in a regression model, residency was the only significant predictor for predicting quality of life in people with moderate dementia after controlling for confounders, including exposure to light level
Onega et al [54], 2016	Light level	—	—	Agitation, depression	CMAI, PAS^y^, BARS^z^, CSDD, DSAOA^aa^, DMAS-17^ab^	—	Those who were exposed to bright light over a 2-week period showed a significant improvement in terms of the levels of depression and agitation compared to those who experienced the placebo bright light exposure
Riemersma-van der Lek et al [55], 2008	Light level	—	—	Agitation, affect, depression, mood, MOSES, overall level of BPSD, standardized scales for cognitive and noncognitive symptoms, limitations of activities of daily living, and adverse effects assessed every 6 months	NPI, MOSES, CSDD, PGCARS^ac^, PGCMS^ad^, CMAI	Actiwatch (supplied by CamNtech)	Light was shown to help reduce depressive symptoms among the participants by 1.5 points on the CSDD; light in combination with melatonin attenuated aggressive behaviors by 3.9 points on the CMAI and increased sleep efficiency by 3.5%
Saidane et al [56], 2023	Light level	—	—	Agitation	CMAI-inspired score	—	Overall, the frequency of agitation-associated behaviors was reduced by 71.2% after the intervention
Sloane et al [57], 2007	Light level, social environment	—	—	Circadian rhythm (rest-activity pattern), sleep	Sleep charts	—	Those exposed to morning and all-day light showed a significant increase in nighttime sleep duration; circadian rhythm showed significant acrophase shifting; effects on daytime sleepiness were inconsistent
Son and Kwag [58], 2020	Noise level, distracted environment	—	—	Anxiety, fear of falling, walking time	State-Trait Anxiety Inventory–Korean Version	—	White noise during walking was shown to positively decrease the state anxiety and fear of falling in walking among older adults with mild dementia
Tartarini et al [59], 2017	Temperature	—	iButton data loggers (Maxim Integrated)	Agitation	CMAI	—	The CMAI scores increased when residents were exposed to relatively cold or warm indoor temperatures at a statistically significant level; the level of agitation was also significantly correlated to the duration of the increased or decreased temperature
Wahnschaffe et al [60], 2017	Light level	—	Spectroradiometer (Specbos 1201; JETI GmbH)	Agitation, circadian rhythm (rest-activity pattern)	CMAI	Actiwatch (supplied by CamNtech)	The comparison of CMAI sumscores between assessments before and during the intervention yielded significant differences with decreased agitated behavior after installation of the dynamic lighting system (*P*<.05) but not for rest-activity patterns
Wahnschaffe et al [61], 2017	Light level, social environment	—	Local weather data	Circadian rhythm (rest-activity pattern)	—	Actiwatch (supplied by CamNtech)	Nocturnal rest, which was proxied by average activity level during five least active hours, was significantly predicted by cloud amount and day length in the highest number of participants (11 out of 20) among all examined rest-activity-related variables, which included interdaily stability, intradaily variability, and relative amplitude

^a^Not applicable.

^b^NPI-Q: Neuropsychiatric Inventory Questionnaire.

^c^CMAI-C: Cohen-Mansfield Agitation Inventory–Community form.

^d^PSQI: Pittsburgh Sleep Quality Index.

^e^CMAI: Cohen-Mansfield Agitation Inventory.

^f^TESS-NH/RC: Therapeutic Environment Screening Survey for Nursing Homes and Residential Care.

^g^NPI: Neuropsychiatric Inventory.

^h^VAS: visual analog scale.

^i^CSDD: Cornell Scale for Depression in Dementia.

^j^MOUSEPAD: Manchester and Oxford Universities Scale for the Psychopathological Assessment of Dementia.

^k^CRBRS: Crichton Royal Behavior Rating Scale.

^l^ABMI: Agitation Behavior Mapping Instrument.

^m^GOME: Group Observational Measurement of Engagement.

^n^NPI-NH: Neuropsychiatric Inventory–Nursing Home version.

^o^SDI: Sleep Disorders Inventory.

^p^QUALID: Quality of Life in Late-Stage Dementia.

^q^PAIN-AD: Pain Assessment in Advanced Dementia.

^r^PEAR: Person-Environment Apathy Rating.

^s^KNPI-Q^:^ Korean version of the Neuropsychiatric Inventory Questionnaire.

^t^CSDD-K: Korean version of the Cornell Scale for Depression in Dementia.

^u^VAS-GV: visual analog scale–global vigor.

^v^VAS-GA: visual analog scale–global affect.

^w^TESS-NH: Therapeutic Environment Screening Survey for Nursing Homes.

^x^MOSES: Multidimensional Observational Scale for Elderly Subjects.

^y^PAS: Pittsburgh Agitation Scale.

^z^BARS: Behavioral Activity Rating Scale.

^aa^DSAOA: Depressive Symptom Assessment for Older Adults.

^ab^DMAS-17: Dementia Mood Assessment Scale, 17 items.

^ac^PGCARS: Philadelphia Geriatric Center Affect Rating Scale.

^ad^PGCMS: Philadelphia Geriatric Center Morale Scale.

### Light

#### Overview

Of the 38 included papers, 34 (89%) examined light as a factor associated with BPSD. Of these 34 studies, 20 (59%) were conducted in North America [24-26,28,31-39,41,43, 47,48,52,54,57], 3 (9%) in Asia [45,50,51], and 11 (32%) in Europe [27,29,30,40,42,46,53,55,56,60,61]. Of the 34 studies, 31 (91%) were conducted in long-term care settings, 2 (6%) were conducted in the participants’ personal residence [25,52], and 1 (3%) was conducted in both the personal residence and the long-term care setting [53]. The median sample size was 63 (IQR 20-122; range 5-326) participants, and the participants’ average age ranged from 73 to 87 years. Light was measured using a variety of light meters or subjective perception scales such as the Therapeutic Environment Screening Survey for Nursing Homes [62] and the environment portion of the Agitation Behavior Mapping Instrument (ABMI) [63].

#### Agitation

Of the 34 studies, 16 (47%) examined the effect of light on agitation either as a syndrome or related to individual agitated behaviors such as wandering or screaming [24-27,29,30,32,35-38,46,54-56,60]. Of these 16 studies, 14 (88%) examined the effect of changing light exposure in RCTs (n=6, 43%) [30,35,37,46,54,55] or quasi-experimental study designs (n=8, 57%) [26,27,29,32,36,38,56,60], while 2 (12%) examined the effect of light on agitated behaviors in a purely observational or natural history study [24,25]. Among the light therapies studied were bright light therapy [30,35,46,54,55] and outdoor environment simulations, such as dawn-dusk simulation [29,56]. A wide range of light intensities was deployed in these light therapies, ranging from 1000 lux [36] to 10,000 lux [54]. The duration and time of day of application of these light therapies were heterogeneous as well; for example, in the study by Onega et al [54], bright light therapies were applied twice a day (once in the morning and once in the afternoon), while in the study by Kolberg et al [46], light therapy was applied from 10 AM to 3 PM with varying light levels before and after this interval. Due to the heterogeneity of the light therapies and study designs, direct comparison across the studies was infeasible. Among these 16 studies, in addition to the multiple approaches to applying light therapy, there was also heterogeneity in the way agitation was assessed. The Cohen-Mansfield Agitation Inventory [64], which measures the frequencies of 29 different agitated behaviors, was the scale used most commonly to assess the level of agitation in people living with dementia across weeks. Other scales used included the Pittsburgh Agitation Scale [65] and the ABMI [63], both of which are based on direct observations of the patient.

The 6 RCTs, which ranged in study duration from 8 weeks to 15 months, used bright light [30,35,37,46,54,55]. However, the results from the RCTs were conflicting. The symptoms of agitation or restlessness were reduced in 3 (50%) of the 6 RCTs [37,54,55] but not in the remaining 3 (50%) [30,35,46]. The lack of improvement in agitation after bright light exposure in these studies [30,35,46] could be due to measurement errors in the subjective assessment of either light level or level of agitation or because of changes in medications not being tracked, as suggested by the authors.

The additional 10 light studies included 8 (80%) quasi-experimental studies [26,27,29,32,36,38,56,60] and 2 (20%) observational studies [24,25]. Of the 8 quasi-experimental studies, 5 (62%) reported that light therapy could reduce agitated behaviors [27,36,38,56,60], while 3 (38%) showed no effect [26,29,32]. Of these 3 studies, 1 (33%) did not yield significant results in part due to a lack of variation in light level [32]. Of the 2 observational studies, 1 (50%) showed that brighter light was associated with wandering [24].

The study by Figueiro et al [38] showed that the effects of light therapy on patients’ agitation varied depending on the participants’ cognitive status. The study, which examined the effect of long-term, all-day exposure to circadian-effective light on the sleep, mood, and behavior in persons with dementia, revealed that the lighting intervention was more effective for people with severe dementia than for those with mild or moderate dementia in reducing agitation [38].

#### Disturbed Sleep and Circadian Rhythm

Of the 17 studies examining the relationship between light and sleep or circadian rhythm [25,29,30,34,36-39,42,45,46, 51-53,57,60,61], 5 (29%) were RCTs [30,37,42,46,52], 5 (29%) were quasi-experimental studies [29,39,45,51,57], 3 (18%) were pretest-posttest studies [36,38,60], 2 (12%) were cross-sectional studies [34,53], and 2 (12%) were case series studies [25,61]. All studies that administered light therapy led to better sleep outcomes such as higher sleep efficiency [36], longer nighttime sleep duration [57], fewer awakenings [51], higher circadian rhythm stability [51], or lower Pittsburgh Sleep Quality Index scores indicating better sleep quality [37].

There can be discrepancies between self-reported sleep quality and objectively measured sleep quality. In the study by Hjetland et al [42], ambient bright light treatment was administered to nursing home patients with dementia in a placebo-controlled RCT. While better sleep was reported using the Sleep Disorders Inventory in the bright light therapy group than in the standard lighting group at weeks 16 and 20, actigraphically measured sleep outcomes showed no statistically significant differences between the 2 groups.

#### Mood-Related Symptoms

Of the 13 studies involving lighting that addressed the relationship between symptoms of depression in people living with dementia and light in the environment [25,30,35-39,41,45-47,54,55], 6 (46%) were RCTs [30,35,37,46,54,55], 4 (31%) were quasi-experimental studies [39,41,45,47], 2 (15%) were pretest-posttest studies [36,38], and 1 (8%) was a case series [25]. A decrease in depressive symptoms was reported in 9 (75%) of the 12 lighting intervention studies [30,36-39,46,47,54,55]; for example, in the RCT described in the study by Onega et al [54], those who were exposed to bright light over a 2-week period showed a significant improvement in depressive symptoms (*P* values ranging from .001 to .017 for DSAOA, DMAS-17, and Cohen-Mansfield Agitation Inventory [CSDD]) while those in the placebo control group showed no improvements in their depressive symptoms. In the quasi-experimental study by Konis et al [47], the participants in a daylight intervention experienced a decrease over the trial in the Cornell Scale for Depression in Dementia (CSDD) score, while the control participants showed a statistically nonsignificant increase in the CSDD score (a higher CSDD score indicates more severe depression). In a pretest-posttest experimental study by Figueiro et al [36], 300 to 400 lux of a blueish-white light was found to significantly decrease depressive symptoms as measured by the CSDD. Some of the studies (2/12, 17%) reported that the efficacy of a lighting intervention is dependent on participants’ characteristics. In the study by Hickman et al [41], analysis indicated a sex-by-treatment interaction. Significant sex differences were found in CSDD scores in response to evening light, all-day light, and standard lighting. Male participants experienced significantly more depressive symptoms under morning light than under standard lighting (*P*=.007). By contrast, female participants experienced fewer depressive symptoms under morning light than under standard lighting, although this result was not statistically significant (*P*=.09).

Similarly, the study by Bromundt et al [29], in which a visual analog scale was used to assess mood, indicated that the improvement in mood from dawn-dusk simulation was dependent on age. In the study, the younger subgroup experienced a stronger positive effect from dawn-dusk simulation on mood [29].

The study by Jao et al [43] examined the effect of light on apathy. The study, which was a case series of people living with dementia in nursing home or assisted living settings, assessed apathy by rating 14 separate 20-minute videos on different days per participant using the Person-Environment Apathy Rating–Apathy subscale [66]. Ambient light (and sound) measurements were collected during the 20-minute video sessions. Light level was found to have had no significant effect on apathy.

The RCT conducted by Kolberg et al [46] in nursing home dementia units with 69 participants examined the effect of light on anxiety and found that the group that received bright light treatment as the intervention had a larger reduction on the composite score of the Neuropsychiatric Inventory–Nursing Home version Affect subsyndrome, which indicated better improvement in depression and anxiety at follow-up after 16 weeks compared to the control group.

### Noise

#### Overview

Of the 11 studies that examined the relationship between noise and dementia-related behaviors, 5 (45%) were experimental studies [31-33,49,58], 5 (45%) were cross-sectional observational studies [24,25,34,40,44], and 1 (9%) was a case study report [43]. Of these 11 studies, 8 (73%) were conducted in North America [24,25,31-34,43,44], 2 (18%) in Asia [49,58], and 1 (9%) in Europe [40]. Of the 11 studies, 10 (91%) were conducted in long-term care settings, and 1 (9%) was conducted in participants’ private home environments [25]. The median sample size was 69 (IQR 38-193; range 10-193) participants, and the participants’ average age ranged from 75 to 86 years. Noise was measured using objective sound level meters in 10 (91%) of the 11 studies, while 1 (9%) study [32] used the environment portion of the ABMI [67].

#### Agitation and Wandering

Of the 5 observational studies, 2 (40%) examined the relationship between noise and agitation among participants with dementia [25,44], and 1 (20%) of the 5 experimental studies aimed to decrease agitation using a white noise intervention [49]. Noise level did not distinguish between episodes of agitation in 1 (50%) [25] of the 2 observational studies, although the study was limited by sample size (10 participants) [25]. In addition, the study by Bankole et al [25] was the only study to examine environmental conditions in the private home setting, which may be distinct from long-term care settings in terms of the effects of noise on agitation. The observational study by Joosse [44], which had 53 participants and used a sound level meter to measure noise levels, found that a higher accumulation of exposure to noise over a day was significantly predictive of agitated behavior in residents with dementia in a long-term care facility [44]. The study by Cohen-Mansfield et al [32] did not find an association between noise and agitation in residents in nursing homes. However, the study was limited by a lack of objective measurement of sound. Finally, in the study by Lin et al [49], an intervention providing daily ambient white noise (55-70 dB) for 20 minutes in the afternoon over a 4-week period significantly decreased the frequency of agitated behavior for participants in the experimental group but not for those in the control group, suggesting that the type of noise, rather than an absence of noise, may be calming.

The observational study by Algase et al [24] examined the effect of noise levels on wandering behavior. In the study of 122 nursing home residents, greater variation in ambient noise level was associated with periods of wandering. There was a significant increase in the risk of wandering (odds ratio 1.09) per SD of variation in ambient noise level [24].

#### Mood-Related Symptoms

The effect of noise on symptoms related to aspects of mood (apathy, affect, or anxiety) was considered in 7 studies that examined associations with depression [25], apathy [31,43], affect or mood [33,34,40], and anxiety [58]: 3 (43%) quasi-experimental studies [31,33,58], 2 (29%) cross-sectional studies [34,40], and 2 (29%) case series studies [25,43]. When noise levels were moderate, rather than low or high, studies found that participants were more highly engaged (ie, less apathetic) [31,34,40] and exhibited greater pleasure or a more positive attitude [33,34]. High levels of background noise in particular were related to less engagement in 2 (67%) of the 3 studies that examined affect or mood [34,40] but were not related to negative affect, leading the authors to conclude that high background noise may cause participants to become less attuned to their environment and bored or sleepy but not uncomfortable or upset [40]. Another study provided evidence of the need for stimulating sound: Jao et al [43] did not find an association between apathy and overall noise level but did find an association between apathy and a lack of clear, discernible sound stimuli (ie, only chaotic background noise). Finally, signs of anxiety in participants with dementia were decreased with an intervention that applied white noise during a walking program in the experimental group only [58].

### Temperature or Humidity

Of the 5 studies that examined the relationship between temperature or humidity and BPSD [24,25,34,40,59], 3 (60%) were cross-sectional studies [24,34,40], and 2 (40%) were case series [25,59]; furthermore, 3 (60%) were conducted in North America [24,25,34], 1 (20%) in Europe [40], and 1 (20%) in Oceania [59]. Of the 5 studies, 4 (80%) were conducted in nursing homes or assisted living units [24,34,40,59], while 1 (20%) was conducted in participants’ private home environments [25]. The median sample size in these studies was 69 (IQR 21-122; range 10-160) participants. The participants’ mean age ranged from 79.7 to 86.6 years.

The case series study by Tartarini et al [59] found that the level of agitation of participants with dementia in a nursing home facility increased significantly when the indoor average temperatures diverged from 22.6 °C. At the same time, the duration of exposure to high temperature (>22.6 °C) and low temperature (<22.6 °C) was linearly correlated with Cohen-Mansfield Agitation Inventory scores. In the cross-sectional studies by Algase et al [24] and Cohen-Mansfield [34], the authors suggested that there was not enough variation in the indoor temperature level or humidity level to identify a relationship with participants’ behaviors.

### Multiple Indoor Environmental Quality Parameters

A total of 8 studies [24,25,31-34,40,43] examined multiple indoor environmental quality parameters (refer to Table 2 for the combination of parameters examined in each study). Of these 8 studies, 7 (88%) were conducted in North America [24,25,31-34,43], and 1 (12%) was conducted in Europe [40]; furthermore, 3 (38%) were quasi-experimental studies [31-33], 3 (38%) were cross-sectional studies [24,34,40], and 2 (25%) were case series [25,43]. While the indoor environmental quality parameters—light level and noise level—were measured in the quasi-experimental studies, they were not manipulated. The intervention used in the quasi-experimental studies involved 9 categories of stimuli not related to the indoor environmental quality parameters.

Of the 8 studies, 5 (62%) reported some associations between indoor environmental quality parameters and BPSD. One of the themes that these studies suggested was that both overstimulation and understimulation from indoor environmental quality parameters can worsen BPSD. Algase et al [24] reported that brighter light and more variations in sound levels were associated with wandering. The studies by Cohen-Mansfield et al [31,33] that examined the effect of the physical environment on pleasure, engagement, and mood had similar findings. It was found that in nursing homes, pleasure was most likely to be experienced by participants with dementia in environments with moderate noise levels [33]. In addition, attention and engagement duration in participants with dementia were higher when light was normal in comparison to a dark room, and their attention and attitude were significantly less positive when the lighting in the room was bright than when the lighting was normal [31]. In the same study, all indicators of engagement significantly favored a moderate level of noise over no noise or low noise levels, and engagement duration was significantly longer for a moderate level of noise compared to high and very high levels of noise.

The cross-sectional study by Garre-Olmo et al [40] measured the physical environment in multiple spaces within the nursing home and found that indoor environmental quality parameters (light, temperature, and noise) in individual living spaces in the nursing home were uniquely associated with participants’ quality of life, behaviors, and mood as measured with the Quality of Life in Late-Stage Dementia Scale; for example, the authors found that low lighting levels in the bedroom were associated with signs of negative affect, high temperature in the bedroom was associated with lower quality of life experienced by the participants, and high noise levels in the living room were associated with low levels of social interaction.

To examine the relationship between multiple environmental factors and agitation in persons with cognitive decline in their home environments, Bankole et al [25] installed environmental sensors in people’s homes that provided continuous data over a 30-day period; in addition, the participants with cognitive decline wore actigraphy devices that provided objective measures of their activity level continuously. Furthermore, 10 caregivers used smartwatches or tablets to report episodes of agitation experienced by the people with cognitive decline in real time, which enabled more direct analysis of the relationship between the behaviors and contemporaneous environmental conditions. Personalized neural networks were built for 6 dyads, which predicted episodes of agitation with light level as a predictor. These networks achieved *F*_1_-score values, which are measures of predictive performance in binary classifications, ranging from 86.12% to 97.07%. *F*_1_-score values can range from 0% to 100%, with a higher *F*_1_-score indicating a better predictive performance. Regarding temperature, the study [25] found that temperature showed strong positive correlation with Teager energy scores from actigraphy device data, which are measures of the aggregate energy of movement [68] and were associated with the agitation exhibited by the people living with dementia.

Of the 8 studies, 3 (38%) failed to identify a link between multiple domains of environmental conditions and BPSD. Jao et al [43] studied the association between apathy in residents with dementia in long-term care facilities and the characteristics of care environments, which included light level, noise level, environmental ambience, crowding, staff familiarity, and environmental stimulation. The authors found that light and sound levels did not show statistically significant effects on apathy [43]. Cohen-Mansfield et al [32] found that lighting and background noise were not associated with levels of agitation in nursing home residents with dementia but that may be due to small variations in these factors. Similarly, Cohen-Mansfield [34] reported no association between noise and mood in people with dementia attending recreational groups.

As seen in Table 2, of the 38 included studies, 6 (16%) relied solely on self-report data for measuring the environment, 13 (34%) used only sensors, and 2 (5%) use both sensors and self-report data, while the environment was not measured but manipulated in 17 (45%) studies. For measurements of BPSD in people living with dementia, of the 38 studies, 36 (95%) used informant-report scales, such as the Neuropsychiatric Inventory Questionnaire; less than half of these studies (n=16, 44%) also used behavioral sensors such as actigraphy to provide objective data.

## Discussion

### Overview

The definition of environment encompasses several major elements: physical environment (eg, light level, noise level, temperature, and humidity), social environment (eg, number of people in proximity), and built environment (eg, furnishings). The focus of this scoping review is on the effects of the indoor environmental quality parameters pertaining to light, noise, temperature, and humidity on BPSD. Given the fact that most older individuals, including people living with dementia, spend most of their time indoors, better understanding of how these physical indoor environmental quality parameters may affect behavioral health is critical. Overall, this review outlines preliminary evidence on the notable linkages between indoor environmental quality parameters and BPSD. Further research remains to be conducted in the field.

### Principal Findings

The evidence base suggests that light during the day is helpful in modulating circadian rhythm, as well as alleviating sleep disturbance and mood-related disorders. Overall, overstimulation and understimulation of environmental factors can lead to challenging behaviors in people living with dementia; for example, when the environment exhibits high levels or low levels of noise, people living with dementia may exhibit more challenging or disturbing behaviors [44,49]. Similarly, challenging behaviors may be associated with the ambient temperatures experienced by the people living with dementia because there was more agitation observed when the environment was either relatively too hot or too cold for the person living with dementia [59]. Such findings are consistent with the existing models that explain the causes of BPSD, including the unmet needs model [69] and the progressively lowered stress threshold model [70]. Ultimately, finding the proper balance of environmental factors for providing the most personal comfort and quality of life for people living with dementia and caregivers may help to minimize the occurrences of BPSD.

### Future Directions of Research

Light is the most researched indoor environmental quality parameter. Most studies on light therapy have shown that it improves sleep and mood in people living with dementia. However, the findings regarding its effect on agitation are conflicting and warrant further research.

The evidence base generally suggests that when noise levels exceed tolerable ranges, behavioral symptoms become more frequent and severe. Studies indicate that both the time duration of exposure to noise and the type of noise exposure could contribute to behavioral symptoms. Future research should explore these factors further.

Studies examining the effects of temperature or humidity on BPSD are largely observational. There exists a need to better understand how thermal comfort affects BPSD in people living with dementia, especially because there are studies suggesting that older adults may need a higher ambient temperature to attain thermal comfort compared to younger adults [71,72].

Environmental factors were often evaluated using self-reported data, which can be prone to biases. The lack of objective measurement of the environment makes measuring and linking the specific effects of the indoor environmental quality parameters on the behaviors of people living with dementia difficult. Of the 38 included studies, 30 (79%) focused on a single environmental factor, measured over relatively short periods of time, limiting the ability to examine more holistically how the environment affects BPSD. To some degree, this single-domain focus may reflect the complexity as well as the cost of deploying multiple sensor types in multiple indoor spaces. As technology advances, indoor environmental quality parameter sensors are expected to become more affordable and scalable.

Although most environmental exposure occurs indoors, studies need to consider in parallel the outdoor environment in terms of its effects on behavior, which is especially important for studies conducted in personal residences because people also spend time outdoors. Going forward, studies should adopt a more holistic approach by deploying sensors to objectively and longitudinally measure the total environment.

While environmental conditions can be objectively sensed and measured, very often, BPSD of the people living with dementia are either reported by the person affected by dementia or observed and interpreted as occurring by an external observer (usually a caregiver) present when the behavior occurs. In this scoping review, 36 (95%) of the 38 included studies relied on these self-reports or observer reports of BPSD. This type of reporting may contain subjective bias. In addition, a variety of scales have been used for assessing the occurrences of BPSD, which differ in their content, structure, and timing of recall. This likely adds to imprecision in comparing the results across multiple studies. More research is needed to examine how different scales for evaluating BPSD relate to each other and, in turn, to multiple environmental conditions. At the same time, whenever possible, the assessment of BPSD should be corroborated with unobtrusive technology such as actigraphy, which can provide objective data.

As evidenced by multiple studies in this review, the effect of the environment on people living with dementia may be dependent on age, sex, and cognitive state. In addition, manifestations of BPSD may be unique for each person living with dementia as well. Recent work by Iaboni et al [73] showed that the features from wearable multimodal sensors varied in their importance to their predictive models for agitation by both individual and behavior type. Finding the optimal environment for each person living with dementia will help support aging in place, which will improve their quality of life and lower the cost of health care. This may be best achieved by being able to examine the environment and behavior at the individual level with a goal of achieving person-centered assessments of well-being in their environment. The use of continuous multimodal sensing data and frequent self-report facilitates the ability to evaluate and analyze data at the individual or “n-of-1” level [73-75], supporting the principle that each individual is unique. Care providers need to know the unique environments, needs, and preferences of each person with dementia to provide the best care and minimize troubling BPSD. Future research should seek to build effective computational models that will incorporate multiple continuous data streams, both environmental and behavioral, that will predict the occurrences of BPSD so that proactive intervention can be implemented [25].

### Implications for Clinical Practice and Policy

At a macro level, the findings of this review, although they are rudimentary, can help inform the design of long-term care facilities and residential homes for people living with dementia. Individual assessments of responses to environmental factors can be conducted upon admission; for example, large windows may be used in long-term care facilities to let in sunlight, and this should be supplemented by a dawn-dusk simulation system to address the excessive, or lack of, sunlight in different seasons to modulate residents’ sleep and circadian rhythm. In addition, the temperature in long-term care facilities and older adults’ homes should be kept at an optimal range that would maximize the comfort of residents. Moreover, quiet flooring and sound-absorbing panels may be used in the long-term care facilities to reduce the noise level; as evidenced by the studies in this review, overly stimulating or noisy environments can increase BPSD. These measures may increase the cost of building and sustaining these long-term care facilities because, for example, maintaining a narrow temperature range can place additional strain on heating, ventilation, and air-conditioning systems in these facilities and may not be energy efficient. However, if the long-term care facilities are more attuned to the needs of people living with dementia, it may result in a decrease in BPSD, improving the quality of life of the people living with dementia and the caregivers as well. This would reduce staff burnout and turnover, ultimately lowering health care costs. Implementing such measures on a broad societal level requires further cost-benefit analysis.

### Limitations

This scoping review has limitations. One of the limitations is that we included studies with heterogeneous designs (eg, observational or experimental), which made the synthesis of results difficult. However, 1 of the aims of this scoping review was to provide an overview of the current state of knowledge regarding the relationship between indoor environmental quality parameters and BPSD. As such, heterogeneous studies were included. Another limitation is that only articles published in English were included. Moreover, only a subset of indoor environmental factors (light level, noise level, temperature, and humidity) were included in this review, while there are other factors relating to BPSD as well, such as psychosocial and biological influences [76]. Notably, this review did not include indoor air quality, another environmental factor that increasingly can be assessed objectively in situ. Air quality has been associated with depression and anxiety in older adults based on spatiotemporal models [77]. A recent study suggested that total volatile organic compound level, among other indoor factors, was significantly correlated with specific areas where behavioral changes occurred in people with moderate to severe dementia in a nursing home [78]. This review only provides evidence for the relationship between a subset of the indoor environmental quality parameters and BPSD, but the relationship likely is best interpreted in a much larger context.

### Conclusions

This review suggests that indoor environmental quality parameters pertaining to light level, noise level, temperature, and humidity may be associated with BPSD. An environment that maximizes the comfort of people living with dementia may decrease their BPSD. Most of the studies (34/38, 89%) in this scoping review pertained to the environmental factor light level, while relatively few studies (5/38, 13%-11/38, 29%) examined the relationship between the remaining indoor environmental quality parameters and BPSD. Among the included studies, there were conflicting findings in the relationship between bright light and agitation, which will need further research. A variety of subjective scales were used to assess the environment and BPSD, which makes synthesizing and comparing results across studies difficult. Going forward, as computational methods and objective sensing technology advance and become more affordable, the behaviors of people living with dementia and their environments should be measured holistically and objectively so that future nonpharmacological intervention can be evidence based.

## Appendix

Multimedia Appendix 1 Embase search strategy for the original search in 2020.

Multimedia Appendix 2 CINAHL search strategy for the original search in 2020.

Multimedia Appendix 3 MEDLINE and PsycINFO search strategy for the original search in 2020.

## Abbreviations

ABMI: Agitation Behavior Mapping Instrument
ADRD: Alzheimer disease and related dementias
BPSD: behavioral and psychological symptoms of dementia
CSDD: Cornell Scale for Depression in Dementia
PRISMA-ScR: Preferred Reporting Items for Systematic Reviews and Meta-Analyses extension for Scoping Reviews
RCT: randomized controlled trial
