# Supplementary material for: Examining the Relationships Between Indoor Environmental Quality Parameters Pertaining to Light, Noise, Temperature, and Humidity and the Behavioral and Psychological Symptoms of People Living With Dementia: Scoping Review
Source: Interact J Med Res. 2024 Aug 9;13:e56452. doi: 10.2196/56452 (PMC11344188; doi:10.2196/56452)
Supplement: Multimedia Appendix 1 [file ijmr_v13i1e56452_app1.docx]

**[
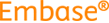
](https://www.embase.com/#search)**

**Embase Session Results**

**No.**

**Query**

**Results**

**641**

#13

(**#9** OR **#11** OR **#12**) AND [humans]/lim AND [english]/lim AND [embase]/lim

**243**

#12

((**alzheim*** OR **dement***) NEAR/7 (**behav*** OR **cognit*** OR **personalit*** OR **mood*** OR **agitat*** OR **emoti*** OR **mental*** OR **disturb*** OR **calm*** OR **upset*** OR **sooth***) NEAR/5 (**environment*** OR **atmospher*** OR **weather*** OR **sound*** OR **nois*** OR **light*** OR **bright*** OR **warm*** OR **hot** OR **hotter** OR **cold*** OR **temperatur*** OR **thermal*** OR **humid***)):ti,ab,de,tn

**216**

#11

**#2** AND **#10**

**9,217**

#10

((**alzheim*** OR **dement***) NEAR/7 (**behav*** OR **cognit*** OR **mood*** OR **agitat*** OR **emoti*** OR **mental***) NEAR/5 (**affect*** OR **caus*** OR **alter*** OR **chang*** OR **increas*** OR **decreas*** OR **lower*** OR **rais*** OR **effect** OR **effects** OR **disturb*** OR **calm*** OR **upset*** OR **sooth***)):ti,ab,de,tn

**2,003**

#9

**#1** AND **#2** AND **#8**

**2,837,912**

#8

**#3** OR **#4** OR **#5** OR **#6** OR **#7**

**644,351**

#7

**'personality'**/exp

**1,023,653**

#6

**'behavior disorder'**/exp OR **'emotion'**/exp

**535,852**

#5

**'quality of life'**/exp OR **'quality of life assessment'**/exp

**17,959**

#4

**'emotional disorder'**/exp

**1,151,701**

#3

**'psychophysiology'**/exp OR **'sleep disorder'**/exp

**647,431**

#2

**'environmental parameters'**/exp

**369,184**

#1

**'dementia'**/exp
