# Supplementary material for: Examining the Relationships Between Indoor Environmental Quality Parameters Pertaining to Light, Noise, Temperature, and Humidity and the Behavioral and Psychological Symptoms of People Living With Dementia: Scoping Review
Source: Interact J Med Res. 2024 Aug 9;13:e56452. doi: 10.2196/56452 (PMC11344188; doi:10.2196/56452)
Supplement: Multimedia Appendix 2 [file ijmr_v13i1e56452_app2.docx]

S22 s19 NOT s20 Expanders - Apply equivalent subjects

Search modes - Boolean/Phrase Interface - EBSCOhost Research Databases

Search Screen - Advanced Search

Database - CINAHL Plus with Full Text 395

S21 S19 AND S20 Expanders - Apply equivalent subjects

Search modes - Boolean/Phrase Interface - EBSCOhost Research Databases

Search Screen - Advanced Search

Database - CINAHL Plus with Full Text 313

S20 (MH "Human") Expanders - Apply equivalent subjects

Search modes - Boolean/Phrase Interface - EBSCOhost Research Databases

Search Screen - Advanced Search

Database - CINAHL Plus with Full Text Display

S19 S12 OR S15 OR S16 OR S17 Expanders - Apply equivalent subjects

Search modes - Boolean/Phrase Interface - EBSCOhost Research Databases

Search Screen - Advanced Search

Database - CINAHL Plus with Full Text Display

S18 S12 OR S15 OR S16 OR S17 Expanders - Apply equivalent subjects

Search modes - Boolean/Phrase Interface - EBSCOhost Research Databases

Search Screen - Advanced Search

Database - CINAHL Plus with Full Text Display

S17 ((alzheim* OR dement*) N7 (behav* OR cognit* OR personalit* OR mood*

OR agitat* OR emoti* OR mental* OR disturb* OR calm* OR upset* OR sooth*)

N5 (environment* OR atmospher* OR weather* OR sound* OR nois* OR light* OR

bright* OR warm* OR hot OR hotter OR cold* OR temperatur* OR thermal* OR

humid*)) Expanders - Apply equivalent subjects

Search modes - Boolean/Phrase Interface - EBSCOhost Research Databases

Search Screen - Advanced Search

Database - CINAHL Plus with Full Text Display

S16 ( ((alzheim* OR dement*) N7 (behav* OR cognit* OR mood* OR agitat* OR

emoti* OR mental*) N5 (affect* OR caus* OR alter* OR chang* OR increas* OR

decreas* OR lower* OR rais* OR effect OR effects OR disturb* OR calm* OR

upset* OR sooth*)) ) AND ( ((environment* OR atmospher* OR weather* OR

sound* OR nois* OR light* OR bright* OR warm* OR hot OR hotter OR cold* OR

humid*) N5 (condition* OR effect* OR impact* OR expos* OR subjected OR

experienc* OR stimula*)) ) Expanders - Apply equivalent subjects

Search modes - Boolean/Phrase Interface - EBSCOhost Research Databases

Search Screen - Advanced Search

Database - CINAHL Plus with Full Text Display

S15 S2 AND S14 Expanders - Apply equivalent subjects

Search modes - Boolean/Phrase Interface - EBSCOhost Research Databases

Search Screen - Advanced Search

Database - CINAHL Plus with Full Text Display

S14 ((alzheim* OR dement*) N7 (behav* OR cognit* OR mood* OR agitat* OR

emoti* OR mental*) N5 (affect* OR caus* OR alter* OR chang* OR increas* OR

decreas* OR lower* OR rais* OR effect OR effects OR disturb* OR calm* OR

upset* OR sooth*)) Expanders - Apply equivalent subjects

Search modes - Boolean/Phrase Interface - EBSCOhost Research Databases

Search Screen - Advanced Search

Database - CINAHL Plus with Full Text Display

S13 ((environment* OR atmospher* OR weather* OR sound* OR nois* OR light*

OR bright* OR warm* OR hot OR hotter OR cold* OR humid*) N5 (condition* OR

effect* OR impact* OR expos* OR subjected OR experienc* OR stimula*))

Expanders - Apply equivalent subjects

Search modes - Boolean/Phrase Interface - EBSCOhost Research Databases

Search Screen - Advanced Search

Database - CINAHL Plus with Full Text Display

S12 S1 AND S2 AND S11 Expanders - Apply equivalent subjects

Search modes - Boolean/Phrase Interface - EBSCOhost Research Databases

Search Screen - Advanced Search

Database - CINAHL Plus with Full Text Display

S11 S3 OR S4 OR S5 OR S6 OR S7 OR S8 OR S9 OR S10 Expanders - Apply

equivalent subjects

Search modes - Boolean/Phrase Interface - EBSCOhost Research Databases

Search Screen - Advanced Search

Database - CINAHL Plus with Full Text Display

S10 (MH "Social Behavior Disorders+") Expanders - Apply equivalent

subjects

Search modes - Boolean/Phrase Interface - EBSCOhost Research Databases

Search Screen - Advanced Search

Database - CINAHL Plus with Full Text Display

S9 (MH "Affect") OR (MH "Affective Symptoms+") OR (MH "Affective

Disorders+") Expanders - Apply equivalent subjects

Search modes - Boolean/Phrase Interface - EBSCOhost Research Databases

Search Screen - Advanced Search

Database - CINAHL Plus with Full Text Display

S8 (MH "Emotions+") Expanders - Apply equivalent subjects

Search modes - Boolean/Phrase Interface - EBSCOhost Research Databases

Search Screen - Advanced Search

Database - CINAHL Plus with Full Text Display

S7 (MH "Sleep Disorders+") Expanders - Apply equivalent subjects

Search modes - Boolean/Phrase Interface - EBSCOhost Research Databases

Search Screen - Advanced Search

Database - CINAHL Plus with Full Text Display

S6 (MH "Mental Processes+") Expanders - Apply equivalent subjects

Search modes - Boolean/Phrase Interface - EBSCOhost Research Databases

Search Screen - Advanced Search

Database - CINAHL Plus with Full Text Display

S5 (MH "Psychophysiologic Disorders+") Expanders - Apply equivalent

subjects

Search modes - Boolean/Phrase Interface - EBSCOhost Research Databases

Search Screen - Advanced Search

Database - CINAHL Plus with Full Text Display

S4 (MH "Psychophysiology+") Expanders - Apply equivalent subjects

Search modes - Boolean/Phrase Interface - EBSCOhost Research Databases

Search Screen - Advanced Search

Database - CINAHL Plus with Full Text Display

S3 (MH "Behavior+") Expanders - Apply equivalent subjects

Search modes - Boolean/Phrase Interface - EBSCOhost Research Databases

Search Screen - Advanced Search

Database - CINAHL Plus with Full Text Display

S2 (MH "Environment+") Expanders - Apply equivalent subjects

Search modes - Boolean/Phrase Interface - EBSCOhost Research Databases

Search Screen - Advanced Search

Database - CINAHL Plus with Full Text Display

S1 (MH "Dementia+") Expanders - Apply equivalent subjects

Search modes - Boolean/Phrase Interface - EBSCOhost Research Databases

Search Screen - Advanced Search

Database - CINAHL Plus with Full Text Display
