# Supplementary material for: Examining the Relationships Between Indoor Environmental Quality Parameters Pertaining to Light, Noise, Temperature, and Humidity and the Behavioral and Psychological Symptoms of People Living With Dementia: Scoping Review
Source: Interact J Med Res. 2024 Aug 9;13:e56452. doi: 10.2196/56452 (PMC11344188; doi:10.2196/56452)
Supplement: Multimedia Appendix 3 [file ijmr_v13i1e56452_app3.docx]

Database: Ovid MEDLINE(R) ALL <1946 to October 01, 2020>

Search Strategy:--------------------------------------------------------------------------------

1 exp Dementia/ (167393)

2 exp environment/ (1296597)

3 exp Radiation, Nonionizing/ (279574)

4 2 or 3 (1430069)

5 exp Behavior/ (1821641)

6 exp psychophysiology/ or exp mental processes/ (1591645)

7 exp sleep wake disorders/ (90408)

8 exp emotion/ (244035)

9 exp affect/ (33581)

10 exp quality of life/ (197501)

11 5 or 6 or 7 or 8 or 9 or 10 (3173371)

12 1 and 4 and 11 (652)

13 ((environment* or atmospher* or weather* or sound* or nois* or light* or bright* or warm* or hot or hotter or cold* or humid*) adj5 (condition* or effect* or impact* or expos* or subjected or experienc* or stimula*)).mp. (397001)

14 1 and 11 and 13 (385)

15 ((alzheim* or dement*) adj7 (behav* or cognit* or mood* or agitat* or emoti* or mental*) adj5 (affect* or caus* or alter* or chang* or increas* or decreas* or lower* or rais* or effect or effects or disturb* or calm* or upset* or sooth*)).mp. [mp=title, abstract, original title, name of substance word, subject heading word, floating sub-heading word, keyword heading word, organism supplementary concept word, protocol supplementary concept word, rare disease supplementary concept word, unique identifier, synonyms] (6741)

16 4 and 15 (78)

17 ((alzheim* or dement*) adj7 (behav* or cognit* or personalit* or mood* or agitat* or emoti* or mental* or disturb* or calm* or upset* or sooth*) adj5 (environment* or atmospher* or weather* or sound* or nois* or light* or bright* or warm* or hot or hotter or cold* or temperatur* or thermal* or humid*)).mp. (244)

18 12 or 14 or 16 or 17 (1133)

19 limit 18 to humans (1001)

20 limit 19 to english language (928)

Database: APA PsycInfo <1806 to September Week 4 2020>

Search Strategy:

--------------------------------------------------------------------------------

1 exp Dementia/ (78140)

2 exp environment/ (201223)

3 exp environmental effects/ (46621)

4 exp illumination/ (8919)

5 2 or 3 or 4 (250809)

6 exp Behavior/ (1735674)

7 exp psychophysiology/ or exp mental processes/ (9526)

8 exp sleep wake disorders/ (19620)

9 exp emotional states/ (290610)

10 exp body awareness/ (1809)

11 exp quality of life/ (43740)

12 6 or 7 or 8 or 9 or 10 or 11 (1939988)

13 1 and 5 and 12 (598)

14 ((environment* or atmospher* or weather* or sound* or nois* or light* or bright* or warm* or hot or hotter or cold* or humid*) adj5 (condition* or effect* or impact* or expos* or subjected or experienc* or stimula*)).mp. (84578)

15 1 and 12 and 14 (231)

16 ((alzheim* or dement*) adj7 (behav* or cognit* or mood* or agitat* or emoti* or mental*) adj5 (affect* or caus* or alter* or chang* or increas* or decreas* or lower* or rais* or effect or effects or disturb* or calm* or upset* or sooth*)).mp. [mp=title, abstract, heading word, table of contents, key concepts, original title, tests & measures, mesh] (4773)

17 5 and 16 (86)

18 ((alzheim* or dement*) adj7 (behav* or cognit* or personalit* or mood* or agitat* or emoti* or mental* or disturb* or calm* or upset* or sooth*) adj5 (environment* or atmospher* or weather* or sound* or nois* or light* or bright* or warm* or hot or hotter or cold* or temperatur* or thermal* or humid*)).mp. (253)

19 13 or 15 or 17 or 18 (972)

20 limit 19 to human (894)

21 limit 20 to english language (854)
